# Restricting retrotransposons: a review

**DOI:** 10.1186/s13100-016-0070-z

**Published:** 2016-08-11

**Authors:** John L. Goodier

**Affiliations:** McKusick-Nathans Institute for Genetic Medicine, Johns Hopkins University School of Medicine, Baltimore, MD USA 212051

**Keywords:** Alu, Autoimmunity, Epigenetics, LINE-1, Methylation, Restriction, Retrovirus, RNAi, SINE, SVA

## Abstract

Retrotransposons have generated about 40 % of the human genome. This review examines the strategies the cell has evolved to coexist with these genomic “parasites”, focussing on the non-long terminal repeat retrotransposons of humans and mice. Some of the restriction factors for retrotransposition, including the APOBECs, MOV10, RNASEL, SAMHD1, TREX1, and ZAP, also limit replication of retroviruses, including HIV, and are part of the intrinsic immune system of the cell. Many of these proteins act in the cytoplasm to degrade retroelement RNA or inhibit its translation. Some factors act in the nucleus and involve DNA repair enzymes or epigenetic processes of DNA methylation and histone modification. RISC and piRNA pathway proteins protect the germline. Retrotransposon control is relaxed in some cell types, such as neurons in the brain, stem cells, and in certain types of disease and cancer, with implications for human health and disease. This review also considers potential pitfalls in interpreting retrotransposon-related data, as well as issues to consider for future research.

## Background

Sixty-five years on from Barbara McClintock’s seminal discovery of mobile DNA [1] we now understand that genomes are dynamic and changeable, with transposable elements (TEs) being major contributors to their fluidity. We recognize that TEs, sometimes called “junk DNA”, are major players in genome evolution and have helped shape the form and function of many genes [2]. Nevertheless, TEs are foremost parasitic DNA, and parasites must be controlled or they will destroy a host. There is far more junk than treasure in mobilomes.

**Fig. 1:**
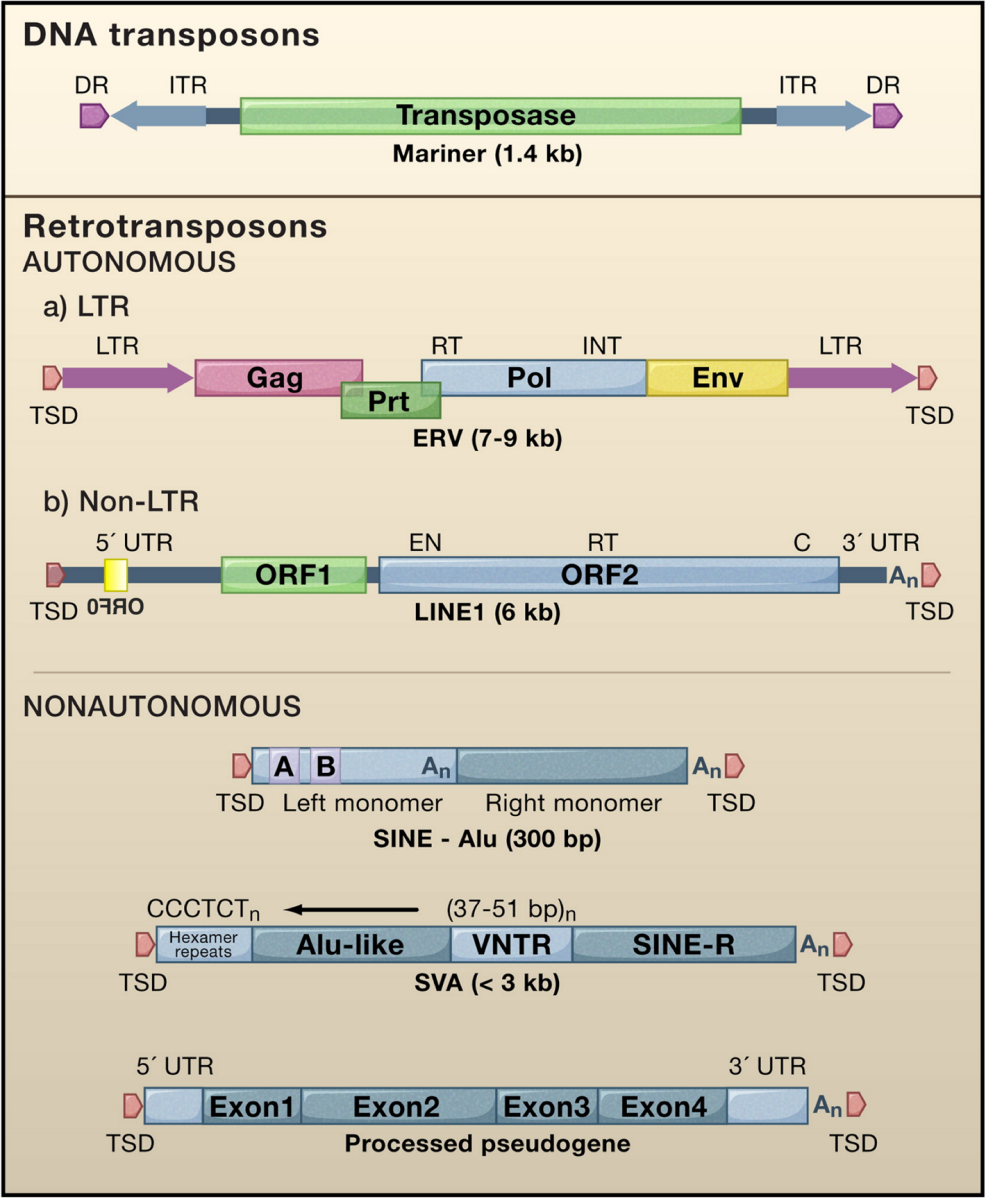
Types of transposable elements in mammals. Abbreviations: DR, direct repeat; ITR, inverted terminal repeat; Gag, group-specific antigen; Prt, protease; Pol, polymerase; Env, envelope; RT, reverse transcriptase domain; INT, integrase domain; TSD, target site duplication; LTR, long terminal repeat; EN, endonuclease domain; C, zinc knuckle domain; A_n_, poly (A); A/B, A- and B-box Pol III promoter; SVA, SINE-R, VNTR, Alu element; VNTR, variable number tandem repeats (reproduced from [3]; Elsevier license number 3803340576977)

DNA transposons comprise about 3 % of the human genome and most move by a “cut and paste” mechanism involving excising an element and reinserting it elsewhere (Fig. 1 [3]). With the exception of at least one family of piggyBac elements in little brown bats [4], no active DNA transposons are known in mammals. There are two classes of retrotransposon. Both move by a “copy and paste” mechanism, involving reverse transcription of an RNA intermediate and insertion of its cDNA copy at a new site in the genome. LTR retrotransposons are named for the long terminal repeats that flank their sequences (reviewed in [5–7]). Endogenous retroviruses (ERVs) are relics of past germline viral infections and for the most part are highly mutated. However, some intracisternal A-particle (IAP) and Etn/MusD family LTR elements remain insertionally active in mice [8], and formation of infective virions by recombination or phenotypic mixing of intact proteins from different ERV proviruses has been reported [9–12]. Among the 31 human endogenous retrovirus subfamilies extant in the human genome, no replication-competent HERVs are known, although their existence has not been ruled out [13, 14], and recently an unfixed fully intact HERV-K (HML2) provirus was identified in some individuals [15]. Many HERVs, and their lone LTRs that populate the genome as a consequence of non-homologous recombination, remain capable of expression and may act as transcriptional regulatory elements for genes (reviewed in [16]).

Non-LTR retrotransposons are as old as the earliest multi-cellular organisms and their 28 clades have origins in the Precambrian Era of 600 million years ago [17, 18]. Long Interspersed Elements (LINEs) and Short Interspersed Elements (SINEs) comprise most of this group in mammals. LINE-1s (L1s), the only currently active autonomous mobile DNA in humans, have been evolving during at least 150 million years of mammalian radiation. Multiple active L1 lineages coexisted in ancestral primates, but for the past 40 Myr there has been a single unbroken lineage of subfamilies [19, 20]. Expansion of L1s was massive, and roughly 500,000 copies now occupy about 17 % of the human genome. Remnant copies of extinct L2 and L3 family elements comprise an additional 4 % [21]. L1s have also been responsible for genomic insertion of 8000 processed pseudogenes and over a million non-autonomous SINEs [22]. B1s and Alus, the predominant SINEs of mice and men, respectively, originate from the 7SL RNA component of the signal recognition particle. Alus are about 300 base pairs in length with a dimeric structure; B1s are monomeric (reviewed in [23]). SVAs are hominid-specific SINEs, and the youngest family of active human retrotransposons. Their name is an acronym reflecting their composite nature: a HERV-K(HML-2)-derived SINE-R, variable-number-of-tandem-repeats (VNTR), and an Alu-like region. There are roughly 2700 SVA copies per human genome, most of which are full-length and about 50 of which may be active [24–31]. SVA-like variants have been described, including a human-specific subfamily generated by fusion of the first intron of the MAST2 gene with an SVA [29, 32, 33], and the LAVA, PVA and FVA elements of non-human primates [34–36].

From 12 Myr ago, the primate LINE-1 expansion slowed, and most insertions are molecular fossils, truncated, rearranged, or mutated [20]. However, although most L1s no longer “jump”, at least 100 remain potentially mobile in any individual diploid human genome [37, 38]. Many more L1s are transcribed. Interestingly, only a small number of the active L1s are ”hot” for retrotransposition and these have accounted for most *de novo* insertions. However, when several of these “hot” Ta-1 L1s were examined across diverse human populations, considerable individual allelic variation affected their ability to retrotranspose [39]. Up to 5 % of newborn children have a new retrotransposon insertion, and to date there are 124 known human disease-causing germline insertions of L1s, Alus, and SVAs [40–42]. The current residual activity of human retrotransposons is the background that escapes a variety of mechanisms that have evolved to limit replication of mobile DNA. This review focuses on mammalian non-LTR retrotransposons and how the cell controls them.

Non-LTR retrotransposons are mobilized by a mechanism very different from that used by retroviruses and LTR retrotransposons. Extensive biochemical analyses of insect R1 and R2 elements, together with genomic sequence analyses, indicate that L1s likely retrotranspose by a process known as target-primed reverse transcription (TPRT) that occurs at the site of DNA insertion. According to this model, L1-encoded endonuclease nicks the bottom strand of target DNA exposing a 3'-hydoxyl that primes reverse transcription of bound L1 RNA. Second-strand DNA synthesis follows and the integrant is resolved in a manner still poorly understood [43]. Short target site duplications (TSDs) of variable length, and occasionally deletions, are generated at new L1 insertion sites.

**Fig. 2:**
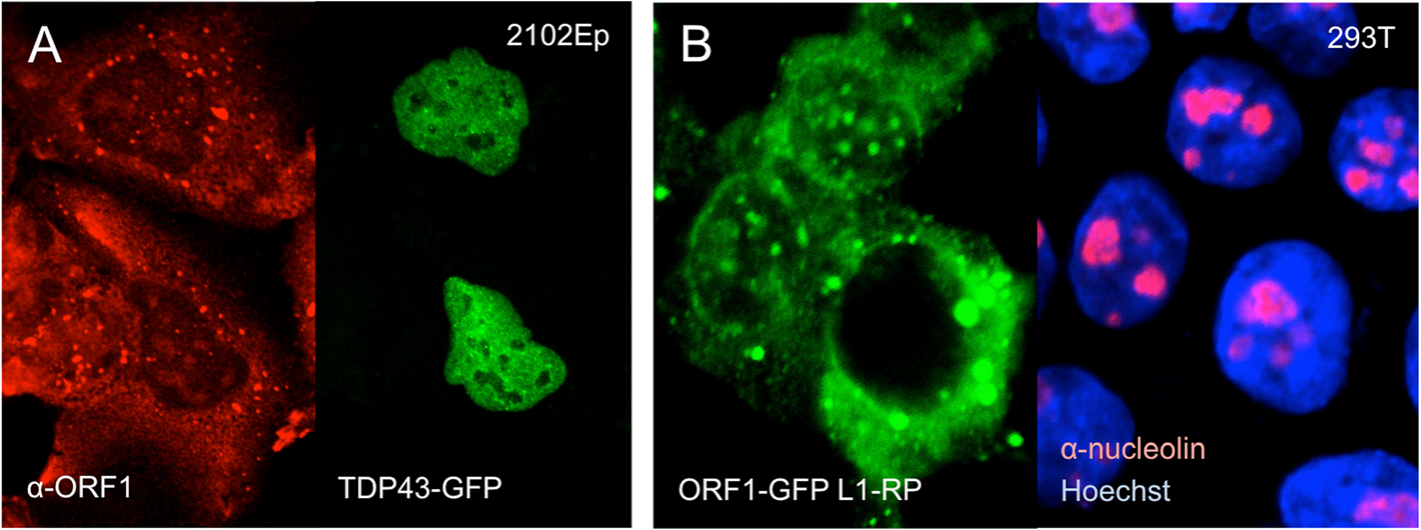
Subcellular distribution of LINE-1 ORF1 protein. **a**. Endogenous ORF1p detected in human embryonal carcinoma 2102Ep cells by a monoclonal antibody [57]. ORF1p is mostly cytoplasmic where it concentrates in SGs and PBs and occasionally at the nuclear membrane. It is faintly detectable in some nuclei and concentrates in nucleoli of a small percentage of cells. Expression of GFP-tagged TDP43 in nuclei but not in nucleoli is shown as a marker. **b**. Exogenously expressed GFP-tagged ORF1p strongly concentrates at the nuclear membrane and in perinucleolar foci of 5 % or fewer human embryonic kidney (HEK) 293T cells, with attendant reduction in size and number of cytoplasmic granules (left panel). Construct ORF1-EGFP L1-RP contains a CMV promoter, ORF1 C-terminally tagged with EGFP, followed by intact downstream L1 sequence. Nucleoli are marked by α-C23 (nucleolin) antibody (Santa Cruz) and nuclei are stained with Hoechst (right panel)

The 6 kilobase bicistronic L1 has a 5' untranslated region (UTR) that functions as an internal promoter, a 3' UTR that ends in a poly (A) signal and tail, and two open reading frames (ORF1 and ORF2) on the sense strand. A weak promoter on the antisense strand of the human 5' UTR [44] lies upstream of a recently identified 216-nt translation-competent ORF0 [45]. Unlike human L1s, mouse L1s have a 5′ UTR consisting of tandemly repeated ∼ 200 bp sequences called monomers [46]. ORF2 encodes a 150 kD protein with endonuclease and reverse transcriptase (RT) activities. While the 40 kD ORF1p RNA-binding protein is essential for LINE-1 retrotransposition, its precise function remains unclear, although it possesses chaperone activity *in vitro* [47, 48]. Early L1 investigations showed ORF1p to be predominantly cytoplasmic where it forms large aggregates, subsequently identified as stress granules (SGs) and processing bodies (PBs) [49–51]. Endogenous L1 RNA has also been detected in PBs [52]. SGs are discrete cytoplasmic aggregates which can be induced by a range of stress conditions, including heat shock, osmotic shock, oxidative stress, viral infection, and overexpression of some proteins. PBs are dynamic cytoplasmic compartments containing molecules involved in mRNA decay and translation inhibition (reviewed in [53, 54]). ORF1p can also concentrate at the perinucleus, is detected faintly in the nucleus, and is seen in nucleoli of a small fraction of cells [55–57] (Fig. 2). Expressed from a full-length L1 construct, ORF1p is present in SGs as a ribonucleoprotein (RNP) complex together with L1 RNA, ORF2p, and many other RNA-binding proteins [58, 59]. Recently, endogenous ORF1p and ORF2p have been reported to also colocalize in nuclear foci of cancer cells [60].

How retrotransposons impact the mammalian cell and genome has been the subject of many other reviews [3, 41, 42, 61–67]. These effects extend beyond simple mutation by genomic insertion. L1 RNA and protein overexpression has been linked with apoptosis, DNA damage and repair, tumor progression, cellular plasticity, and stress response [68–72]. Consequently, the cell has evolved a battery of defenses to protect against the dangers of unfettered retrotransposition. It is not surprising that many of the known anti-retrotransposon restriction factors are also anti-retroviral. Phylogenetic analyses suggest that eukaryote non-LTR retrotransposons predate LTR retrotransposons, which in turn gave rise to retroviruses through the acquisition of an envelope (env) gene [73–76]. Indeed, some restriction factors may have first evolved to control ancient endogenous retroelements and were later recruited to the fight against exogenous invaders. It is reasonable to presume that from the study of factors controlling endogenous retrotransposition new insights into the control of viral infections will emerge.

Until recently, our knowledge of the cellular factors that interact with mammalian retrotransposons to facilitate or frustrate their activity lagged behind our understanding of such factors in yeast and flies [77–80]. Nevertheless, in recent years, with the aid of mouse transgenic models, improved antibodies, efficient strategies for immunoprecipitating retrotransposon RNP complexes from cells, new high-throughput (HT) DNA resequencing strategies, and cell culture retrotransposition assays, we have significantly increased our understanding of how the mammalian cell attempts to coexist with a molecular parasite whose unchecked activity could be bad news indeed.

**Fig. 3:**
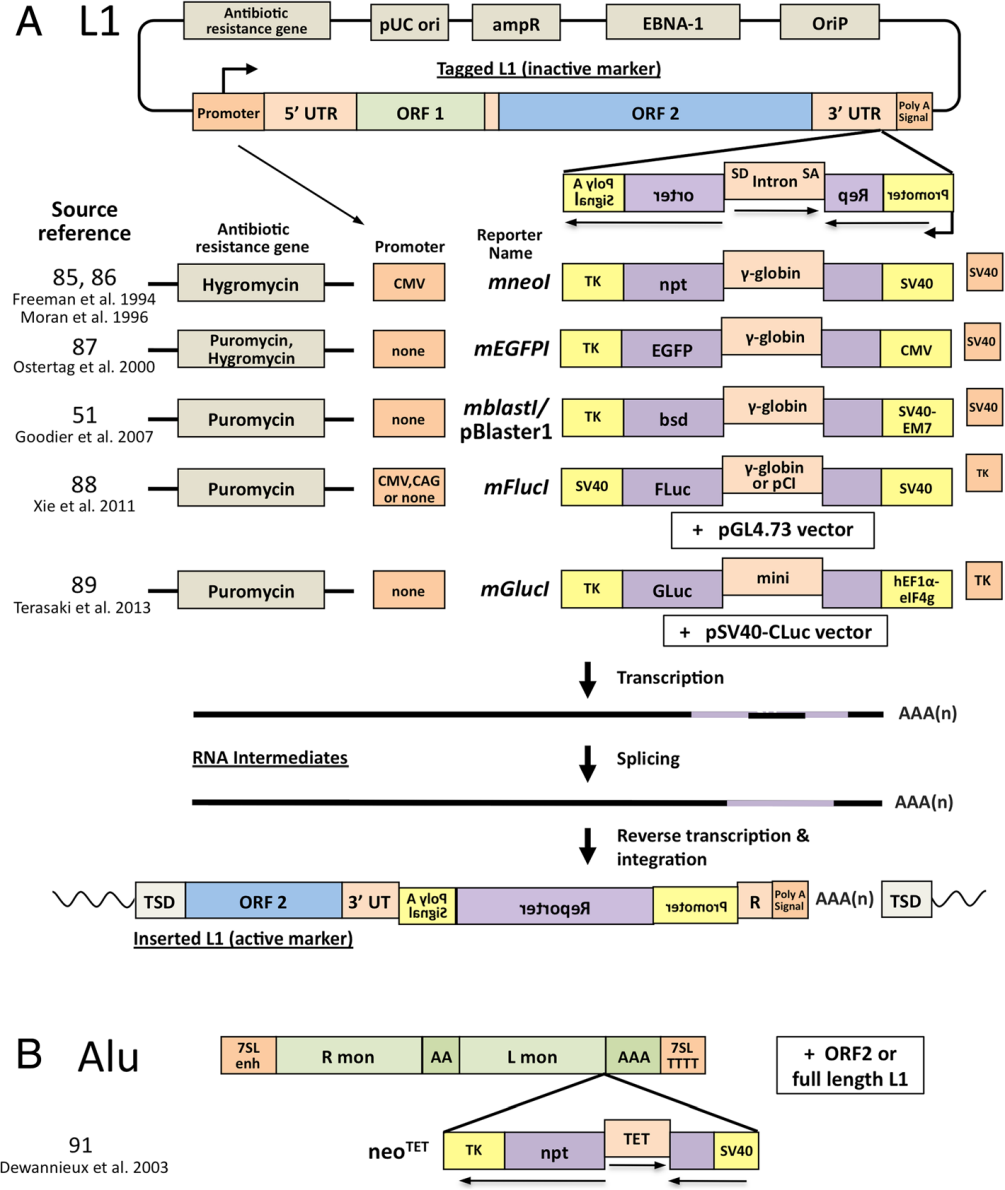
Cell culture retrotransposition assay reporter gene cassettes come in a variety of flavors. **a**. LINE-1 assays. A retrotransposition-competent L1 and reporter cassette is cloned in pCEP4 (Invitrogen)-based vectors, which encode EBNA-1 and OriP and so replicate in primate cells. Variants of the vectors also contain or lack an exogenous promoter upstream of the L1, and encode resistance to hygromycin or puromycin permitting antibiotic selection of transfected cells. *mneoI* and *mblastI* reporter cassettes confer drug resistance to cells having a retrotransposition event. These cells are expanded in culture to form colonies, fixed, stained, and the number of colonies scored. The *mEGFPI* cassette fluorescently marks cells with retrotransposon insertions and allows their numbers to be counted by flow cytomentry. Firefly luciferase gene *mFlucI* reporter vectors may be cotranfected with pGL4.73 (Promega) or other vector which constitutively expresses renilla luciferase from transfected cells. Following cell lysis, retrotransposition levels, indicated by firefly luciferase, are adjusted to renilla expression to control for differences in transfection efficiency. The *mGlucI* cassette expresses Gaussia luciferase which when secreted into the media serves as an effective read-out of accumulated retrotransposition events. Levels of Gluc may be normalized to those of Cypridina luciferase (which is also secreted and does not cross-react with Gluc) constiitutively expressed from the cotransfected pSV40-CLuc vector (NEB). Simply by sampling small aliquots of cell culture media, retrotransposition may be assessed in a single well at multiple time points without cell lysis. Luciferase-based reporter cassettes are amenable to HT retrotransposition screening. **b**. The Alu assay. An active Ya5 Alu and neo^TET^ cassette interrupted by a *Tetrahymena thermophila* self-splicing 23S rRNA Group I intron is cloned between the 7SL pol III enhancer and terminator. When this construct is co-expressed with L1 ORF2 alone or a full-length retrotransposition-competent L1, Alu RNAs are reverse transcribed along with the spliced *npt* gene and integrated into the genome to confer neomycin resistance. Abbreviations: 7SL enh, 7SL enhancer; 7SL TTTT, 7SL transcription terminator; ampR, ampicillin resistance gene; bsd, blasticidin S deaminase gene; CMV, cytomegalovirus promoter; EBNA-1, Epstein-Barr nuclear antigen 1; EGFP, enhanced green fluorescent protein; L mon, left monomer; mini, chimeric mini-intron of the plasmid psiCHECK-2 (Promega); npt, neomycin phosphotransferase gene; oriP, latent origin of replication; pCI: synthetic intron from pCI (Promega); R mon, right monomer; SA, splice acceptor; SD, splice donor; SV40, simian virus 40 early enhancer/promoter; TET, *T. thermophila* self-splicing intron; TK, herpes simplex virus thymidine kinase poly(A) signal

Of all the tools in the toolbox of mammalian retrotransposon research, after twenty years the cell culture assay for retrotransposition remains the most important (Fig. 3; reviewed in [81, 82]). It built upon earlier assays that tracked Ty1 LTR retrotransposition in budding yeast [83]. A reporter gene cassette, interrupted by a backwards intron and inserted in opposite transcriptional orientation into the 3' UTR of a retrotransposition-competent L1, is expressed only when the L1 transcript is spliced, reverse-transcribed, its cDNA inserted in the genome, and the reporter gene expressed from its own promoter. The original neomycin phosphotransferase gene reporter [84–86] was later joined by enhanced green fluorescent protein, blasticidin S-resistance, firefly luciferase, and secreting gaussia luciferase gene constructs [51, 87–89] (Fig. 3). Alu, SVA, and mouse SINE non-LTR, and IAP and HERV LTR retrotransposition assays have also been established [10, 90–96]. While immensely effective in revealing *cis*- and *trans*-acting factors of retrotransposition, the degree to which these plasmid-based assays truly reflect endogenous levels of retrotransposition is often uncertain. Fortunately, cell culture results can now be confirmed by HT genome sequencing [97].

## Lines of defense

To a significant degree, non-LTR retrotransposon sequence itself and the nature of TPRT mitigate genomic insertions. Most L1s die at the time of TPRT, undergoing 5' truncations or inversions, or internal deletions. Most of the insertions that remain intact ultimately lose their ability to remobilize due to DNA recombination or mutation [98]. It has also been suggested that the adenosine richness of the L1 template retards processivity of transcription and limits retrotransposition [99]. Mutations to binding sites for transcription/enhancer factors, including E2F1/RB1, ETS, p53, RUNX3, SOX2, SP1, TCF-LEF, and YY1 for L1s and AHR, CTCF, RAR and SLUG for mouse SINEs, modulate TE expression and in some cases retrotransposition [100–116] (All factors and their full names are listed in Table 1). Cryptic splice sites in L1 RNA transcripts induce a complex pattern of splicing that may remove portions of the ORFs or the 5' UTR [117, 118]. Alus also contain cryptic splice sites, and when resident in genes are frequently exonized into mRNA transcripts and are occasionally translated [119–122]. Interestingly, heterogeneous nuclear ribonucleoprotein C (HNRNPC) protects the cell from Alu-mediated aberrant exonization by competing with splicing factor U2AF2 for binding at Alu splice sites [123]. Alu lacks its own Pol III transcription termination signal, requiring termination at signals in downstream flanking DNA with possible loss of retrotransposition efficiency. The L1 possesses a poly (A) termination signal that is inherently weak and permits occasional read-through of L1 transcripts, necessitating their termination at signals downstream. Interestingly, *in silico* studies show that approximately 15 % of L1s have transduced 3’ flanking DNA to a new genomic location, in the process generating between 19 and 30.5 Mb of new DNA or as much as 1 % of the human genome [124–127]. Cryptic polyadenylation signals are also scattered along the A-rich length of the L1, and consequently a majority of L1 RNAs are prematurely truncated and incapable of forming functional RNPs [99, 128]. Post-translational protein modifications, including phosphorylation of ORF1p [129, 130], may also modulate retrotransposition.

**Table 1:** Cellular factors associated with mammalian non-LTR retrotransposon activity

**Protein Symbol (Hs) (1)**	**Alternative Name (Hs or Mm) (1)**	**Protein Name**	**Source species of factor (1)**	**Comment**	**Altered retrotransposition reported**	**References**
**Post-transcriptional**						
AICDA	AID	Activation-induced cytidine deaminase	various	RNA-editing cytidine deaminase	Y	[142, 144]
APOBEC1		Apolipoprotein B mRNA editing enzyme catalytic subunit 1	various	RNA-editing cytidine deaminase	Y	[143, 145]
APOBEC3		Apolipoprotein B mRNA editing enzyme catalytic subunit 3	Hs	RNA-editing cytidine deaminase	Y	[136, 145, 149, 152, 153, 160, 515–521]
ATG5		Autophagy related 5	Hs	Autophagy E1-like activating enzyme	Y	[52]
BECN1	ATG6	Beclin 1	Mm	Autophagy factor	Y	[52]
CALCOCO2	NDP52	Calcium binding and coiled-coil domain 2	Hs	Macroautophagy receptor	Y	[52]
HNRNPL		Heterogeneous nuclear ribonucleoprotein L	Hs		Y	[56, 236, 240, 261]
KIAA0430	MARF1, LKAP	Meiosis arrest female protein 1	Hs	Regulator of oogenesis		[266, 267]
MOV10		Mov10 RISC complex RNA helicase	Hs	Putative ATP-dependent RNA helicase	Y	[232, 234–237]
MTNR1A	MT1	Melatonin receptor 1A	Hs		Y	[258]
PABPC1	PABP1	Poly(A) binding protein cytoplasmic 1	Hs		Y	[259]
RNASEL		Ribonuclease L	Hs	Endoribonuclease/component of 2-5A system	Y	[254]
SAMHD1		SAM and HD domain containing deoxynucleoside triphosphate triphosphohydrolase 1	Hs,Mm		Y	[168, 169, 171, 172]
SQSTM1	P62	Sequestosome 1	Hs	Macroautophagy receptor	Y	[52]
TEX19	TEX19.1	Testis expressed 19	Mm			[264, 265]
TREX1	AGS1	Three prime repair exonuclease 1	Mm	3' exonuclease	Y	[257]
ZC3HAV1	PARP13, ZAP	Zinc finger CCCH-type containing, antiviral 1	Hs	Antiviral protein	Y	[236, 240, 250]
**piRNA/RNAi-Pathways**						
ASZ1	GASZ	Ankyrin repeat, SAM and basic leucine zipper domain containing 1	Mm			[206, 217]
DDX4	MVH, VASA	DEAD-box helicase 4	Mm	ATP-dependent RNA helicase		[210, 217]
DGCR8	pasha	DGCR8 microprocessor complex subunit	Hs	Subunit of microprocessor	Y	[196, 197]
DICER1	DCR1	Dicer 1, ribonuclease type III	Hs	dsRNA endoribonuclease	Y	[191, 194]
DROSHA	RNASEN	Drosha ribonuclease type III	Hs	dsRNA-specific endoribonuclease/subunit of microprocessor	Y	[197]
EXD1		Exonuclease 3'-5' domain containing 1	Mm	3'-5' exonuclease activity		[223]
FKBP6		FK506 binding protein 6	Mm	Cis-trans peptidyl-prolyl isomerase		[215]
GTSF1	CUE110	Gametocyte specific factor 1	Mm	UPF0224 (FAM112) family member		[209]
HENMT1		HEN1 methyltransferase homolog 1	Mm	2'-O-methylation pf piRNAs		[221]
HSP90AA1	HSP90N	Heat shock protein 90 alpha family class A member 1	Mm	Molecular chaperone		[220]
MAEL		Maelstrom spermatogenic transposon silencer	Mm			[204]
MIR128-1		microRNA 128-1	Hs		Y	[192]
MOV10L1		Mov10 RISC complex RNA helicase like 1	Mm	Putative ATP-dependent RNA helicase		[244, 245]
	Nct1/2	Non-coding in testis 1/2	Mm	piRNA encoding non-coding RNAs		[205]
PIWIL1	MIWI	Piwi like RNA-mediated gene silencing 1	Mm	Argonaute family member		[212]
PIWIL2	MILI	Piwi like RNA-mediated gene silencing 2	Mm	Argonaute family member		[200, 211, 216, 217]
PIWIL4	MIWI2	Piwi like RNA-mediated gene silencing 4	Mm	Argonaute family member		[202, 203, 211, 212, 222]
PLD6	MITOPLD	Phospholipase D family member 6	Mm			[213]
TDRD1		Tudor domain containing 1	Mm			[207]
TDRD5		Tudor domain containing 5	Mm			[214]
TDRD9		Tudor domain containing 9	Mm	Putative ATP-dependent RNA helicase		[208, 217]
TDRD12		Tudor domain containing 12	Mm	Putative ATP-dependent RNA helicase		[218]
TDRKH	TDRD2	Tudor and KH domain containing	Mm			[219]
**Epigenetic/Nuclear Factors**						
ALKBH1		alkB homolog 1, histone H2A dioxygenase	Mm	3-methylcytosine demethylase		[366]
ATM		ATM serine/threonine kinase	Hs,Mm	PI3/PI4-kinase family member	Y	[369–371]
CHAF1	CAF1	Chromatin assembly factor 1	Mm	Assembles histone octamer		[297]
DCLRE1C	ARTEMIS	DNA cross-link repair 1C	Gg	Roles in NHEJ DNA repair and V(D)J recombination	Y	[367]
DNMT1		DNA methyltransferase 1	Hs,Mm			[313, 340]
DNMT3A		DNA methyltransferase 3 alpha	Hs,Mm			[313, 340, 342, 344]
DNMT3B		DNA methyltransferase 3 beta	Hs,Mm			[313, 340, 342, 344]
DNMT3L		DNA methyltransferase 3 like	Mm	DNA methyltransferase cofactor		[222, 342, 345]
EHMT2	G9A	Euchromatic histone lysine methyltransferase 2	Mm	Histone H3K9me1 and H3K9me2 methyltransferase		[289]
ERCC1	RAD10	ERCC excision repair 1, endonuclease non-catalytic subunit	Cg	Nucleotide excision repair	Y	[368]
ERCC4	XPF	ERCC excision repair 4, endonuclease catalytic subunit	Hs,Cg	Nucleotide excision repair (heterodimer with ERCC1)	Y	[368]
KDM1A	LSD1	Lysine demethylase 1A	Mm	Histone H3K4me and H3K9me demethylase		[363]
LIG4		DNA ligase 4	Gg	Roles in NHEJ DNA repair and V(D)J recombination	Y	[367]
MECP2		Methyl CpG binding protein 2	Hs,Mm	Binds methylated DNA	Y	[355–357]
MORC1		MORC family CW-type zinc finger 1	Mm	Role in early spermatogenesis		[352]
PRKDC	XRCC7, DNA-PKcs	Protein kinase, DNA-activated, catalytic polypeptide	Cg	NHEJ DNA double-strand break repair	Y	[68, 72]
SIRT6		Sirtuin 6	Mm	NAD-dependent protein deacetylase	Y	[473]
SUV39H		Suppressor of variegation 3–9 homolog 1	Hs,Mm	Histone H3-K9 methyltransferase 1		[286, 288, 291]
TRIM28	KAP1	Tripartite motif containing 28	Hs,Mm	Nuclear corepressor for KRAB-ZFPs		[313]
UHRF1	NP95, ICBP90	Ubiquitin-like with PHD and ring finger domains 1	Mm	RING-finger type E3 ubiquitin ligases		[351]
XRCC4		X-ray repair cross complementing 4	Cg	DNA single-strand break repair	Y	[68, 72]
XRCC6	KU70	X-ray repair cross complementing 6	Gg	ssDNA-dependent ATP-dependent helicase	Y	[367]
**Krüppel-associated box domain-containing zinc finger proteins (KRAB-ZFPs)**						
	GM6871	Predicted gene 6871	Mm			[313]
ZBTB16	PLZF	Zinc finger and BTB domain containing 16	Hs,Mm		Y	[317]
	ZFP819	Zinc finger protein 819	Mm			[314]
ZNF91		Zinc finger protein 91	Hominidae			[315]
ZNF93		Zinc finger protein 93	Hominidae		Y	[315]
**Other transcription factors**						
AHR		Aryl hydrocarbon receptor	Mm,Dr	Ligand-activated transcription factor		[109, 114]
CTCF		CCCTC-binding factor	various	BORIS + CTCF gene family member		[108, 116]
ETS1		ETS proto-oncogene 1, transcription factor	Hs	ETS transcription family member		[102]
RAR		Retinoic acid receptor	Hs	Thyroid-steroid hormone receptor superfamily member		[107, 115]
	RB/E2F1	RB transcriptional corepressor proteins/E2F transcription factor 1	Hs,Mm	Transcription repressor complex		[112, 298]
RUNX3		Runt-related transcription factor 3	Hs	Runt domain-containing transcription family member	Y	[104]
SNAI2	SLUG	Snail family transcriptional repressor 2	Mm,Dr	Snail C2H2-type zinc finger transcription family member		[109, 114]
SOX2		SRY-box 2	Hs,Mm	SRY-related HMG-box (SOX) transcription family member		[103, 106, 111]
SP1		Sp1 transcription factor	Hs	zinc finger transcription factor		[102]
	TCF-LEF	T-cell factor/lymphoid enhancer factor	Rn	Wnt transcription factors		[111]
TP53	p53	tumor protein p53	Dr,Hs,Mm	tumor suppressor protein	Y	[110, 299, 300]
YY1		YY1 transcription factor	Hs,Mm	GLI-Kruppel zinc finger transcription family member		[101, 105, 113]

(1) Cg, *Cricetulus griseus*; Cl, *Canis lupus*; Dr, *Danio rerio*; Gg, *Gallus gallus*; Hs, *Homo sapiens*; Mm, *Mus musculus*, Rn, *Rattus norvegicus*

The cell has also evolved a phalanx of trans-acting restriction factors that function as an early defense against both viral infection and endogenous retroelements. Many of these proteins are involved in nucleic acid metabolism and may be constitutively expressed or induced, often by type I interferons. Typically they form a rapid response to infection, and act in the cytoplasm. Early examples were found by comparing cell lines that were permissive or restrictive for viral infection.

### Apolipoprotein B mRNA editing enzyme, catalytic polypeptide-like (APOBEC)/ Activation-induced cytidine deaminase (AID) proteins

The first anti-retrotransposon restriction factors identified were AID/APOBEC proteins, an evolutionarily conserved, vertebrate-specific family of cytidine deaminases. While rodents have a single APOBEC3 family member, humans have seven APOBEC3s (A3A-D, A3F, A3G and A3H). It was discovered that A3G is packaged into virions of Vif-deficient HIV-1, where during reverse transcription it deaminates cytosines to uracils in the nascent first-strand HIV cDNA. Uracils in the cDNA cause dG > dA hypermutations during second strand synthesis, limiting viability of the viral progeny [131–133]. In the past 10 years, A3B and A3F have also been shown to have antiretroviral activity, and some APOBEC3 proteins are effective against other classes of virus (reviewed in [134]).

The discoveries that AID/APOBEC proteins restrict not only infecting viruses but also LTR and non-LTR retrotransposons have been summarized in previous reviews [135–141]. All APOBEC3 proteins inhibit LINE-1 retrotransposition to varying degrees, with A3A and A3B being most effective. AID and APOBEC1 proteins both inhibit cell culture L1 and LTR element retrotransposition [142–145]. AID may also promote methylation of TEs in the nuclei of primordial germ cells (PGCs) [146]. Interestingly, Khatua et al. [147] revealed a way in which restriction may be transferred from one cell to another, showing that extruded exosome vesicles can encapsulate A3F and A3G mRNAs and be taken up by other cells to inhibit their ability to support Alu and L1 retrotransposition. Tumor-derived microvesicles are also enriched in LINE-1, Alu, and especially HERV RNAs [148]. Apart from being a potential conduit for moving TEs between cells, these tumor-derived microvesicles may make useful cancer biomarkers if they can be confidently detected in human blood or sera.

Unexpectedly, catalytically inactive APOBEC3s still inhibit non-LTR retrotransposons, and several investigations found scant genomic evidence for L1 editing by cytidine deamination [149–151]. Deamination-independent mechanisms of APOBEC action were therefore proposed, including sequestration of retrotransposon RNPs in high molecular weight cytoplasmic complexes and their targeting to SGs and PBs for possible degradation by RNAi silencing [152–158]. However, *in silico* analyses by Carmi et al. [159] confirmed extensive editing of LTR retrotransposons and found strong evidence for editing of SVAs (20 %) and mouse L1 elements (0.74 %), but minimal editing of human L1s (which occurred mostly within older subfamilies). Richardson et al. [160] then proposed that annealed L1 RNA normally protects first-strand cDNA from deamination, but that transiently exposed single-stranded (ss) cDNA occurring during TPRT becomes accessible to deamination by A3A. Normally the cell repairs U mutations, but by inhibiting uracil DNA glycosylase in cell culture, these authors detected A3A-induced L1 mutations. Moreover, overexpression of both A3A and RNase H, which degrades RNA:DNA hybrids, increased L1 cDNA mutation in an *in vitro* RT assay [161]. HIV, unlike L1, encodes RNase H activity, which may make its cDNA more susceptible to APOBEC3-mediated deamination.

### SAM domain and HD domain 1 (SAMHD1)

Another important member of the anti-retroviral arsenal is SAMHD1, a dGTP-activated deoxynucleoside triphosphate triphosphohydrolase. It has been proposed that SAMHD1 degrades the dNTP pool in non-dividing cells to levels below that necessary for reverse transcription of retroviruses and replication of some DNA viruses [162–166]. Loss of SAMHD1 has been linked to Aicardi-Goutières syndrome (AGS), an early-onset inflammatory disorder affecting particularly the brain [167].

Overexpression of SAMHD1 inhibits, while coexpression of SIV-encoded accessory protein viral protein X (Vpx) or depletion of endogenous SAMHD1 increases non-LTR retrotransposition in cell culture. Seven of eight AGS-related mutations in SAMHD1 reduced inhibition of cell culture LINE-1 retrotransposition by 40 % or more [168]. On the other hand, nine naturally occurring polymorphisms failed to alter SAMHD1 inhibition of retrotransposition [169]. One might expect patients with mutant SAMHD1 alleles to show increased retrotransposition; however, sequencing of bulk tissue and single neurons from the brain of one AGS patient revealed no increase of L1 insertions compared with controls [170].

Although SAMHD1 restricts HIV and SIV in non-dividing cells only, non-LTR retrotransposition is reduced in dividing cells where dNTPs are constantly replenished. Furthermore, SAMHD1 proteins with mutations in the NTPase catalytic domain or at a residue whose phosphorylation is important for retroviral restriction still inhibit cell culture retrotransposition [168] (although Hu et al. [171] reported an NTPase mutant that failed to inhibit retrotransposition). Tetramer formation by SAMHD1 is required for both dNTPase activity and regulation of HIV-1 and LINE-1s [172].

These data predict a mechanism other than dNTPase activity for restricting L1s. SAMHD1 also possesses ribonuclease activity, which even in the absence of functional dNTPase inhibits HIV-1 replication [173]: its effect on retrotransposons remains to be tested. Zhao et al. [168] reported that SAMHD1 reduced L1 reverse transcription by inhibiting ORF2p but not ORF1p. Hu et al. [171] proposed a novel mechanism whereby SAMHD1 enhances assembly of cytoplasmic stress granules that then sequester L1 RNPs and prevent their retrotransposition. Depletion of SG proteins G3BP1 (which binds the L1 RNP) or TIA1 prevented SG formation and reduced SAMHD1 inhibition of LINE-1s. While LINE-1 proteins and RNA concentrate in SGs and PBs along with factors linked with their restriction, a direct role for cytoplasmic granules in modulating retrotransposition remains unclear. Previous experiments investigated PBs and LTR retrotransposons only, and results were conflicted. PBs were required for yeast Ty1 and Ty3 virus-like particle (VLP) assembly and retrotransposition [174–176], but PBs inhibited mouse IAPs [157]. It remains to be determined if cytoplasmic aggregates are a retrotransposition dead-end or an integral part of the L1 life cycle.

### RNA-induced Silencing Complex (RISC) and Piwi-interacting RNA (piRNA) pathway proteins

Small interfering RNA (siRNA)-mediated post-transcriptional gene silencing is an ancient strategy for limiting the spread of mobile genetic elements. RNA interference (RNAi) can act at the post-transcriptional level by causing RNA degradation and loss of translation, or at the transcriptional level by inducing epigenetic modifications. Several lines of evidence suggest a direct role for small RNAs in mammalian retrotransposon silencing (reviewed in [177–183]. A large number of endogenous retrotransposon-related small RNAs of a size consistent with siRNAs, miRNAs and piRNAs have been detected in cells [179, 184–188] (reviewed in [189]). Treating cells with *in vitro* diced L1 siRNAs hindered cell culture retrotransposition [190], and L1-related endo-siRNAs decreased retrotransposon activity, apparently by promoter hypermethylation [191]. Recently, a specific microRNA, mir-128, was found to bind L1 RNA and repress its integration in HeLa and induced pluripotent stem cells (iPSCs) [192]. Indeed, it has been proposed that miRNAs originally evolved from TEs [189]. The question remains, however, as to whether RNAi pathways evolved to silence TEs themselves or gene transcripts that happened to contain target TE sequences [193].

In the nucleus, DGCR8 binds DROSHA, an RNase III-type enzyme, to form the Microprocessor complex. Microprocessor cleaves primary miRNAs (pri-miRNAs), which are then further processed in the cytoplasm to mature miRNAs by DICER and loaded into Argonaute (AGO)-containing RISCs. Knockdown of DICER1 (which also processes siRNAs from dsRNAs) or AGO2 causes an increase in the rate of retrotransposition of tagged L1s in cell culture [191, 194]. Elevated transcription of murine L1 and IAP elements has been observed in embryonic stem cells (ESCs) of *Dicer*-null mice [195]. Interestingly, DGCR8 also directly binds L1- and SINE-derived RNAs, presumably at hairpin structures, which are apparently cleaved by Microprocessor in a manner independent of DICER and miRNAs. Both DROSHA and DGCR8 affect cell culture retrotransposition [196–198]. Non-LTR retrotransposon RNAs that escape Microprocessor surveillance in the nucleus may be captured in the cytoplasm for further processing by DICER and RISC loading.

 piRNAs are small RNAs slightly longer than siRNAs (24–30 nt) that are processed independently of DICER and silence TEs specifically in the germline. They mediate both PIWI protein endonuclease-slicer activity [199] and *de novo* methylation of TE sequences (discussed below). A large proportion of mouse prepachytene piRNAs derives from retrotransposon sequences [200–202], and the importance of piRNA pathway proteins in repressing retrotransposons in prenatal gonad development and spermatogenesis has repeatedly been demonstrated in mutant mouse lines. Loss of EXD1, FKBP6, GASZ/ASZ1, GTSF1, HENMT1, HSP90α, MAEL, MILI/PIWIL2, MIWI/PIWIL1, MIWI2/PIWIL4, MVH/DDX4, PLD6/MITOPLD, TDRD1, TDRD5, TDRD9, TDRD12, or TDRKH/TDRD2 protein, or the piRNA-encoding non-coding RNAs Nct1/2 is accompanied by derepression of LINE-1 and IAP retrotransposons [201–223]. These studies generated much discussion in the RNAi and retrotransposon fields. However, they failed to provide a crucial piece of information: do the observed accumulation of retrotransposon RNAs and proteins mean increased numbers of endogenous insertion events? Also, it remains to be determined if increased retrotransposition contibutes to the male germline defects and sterility observed in many of these knockout (KO) mice. With the advance of HT genome sequencing, this information can now be obtained.

### Moloney leukemia virus 10, homolog (mouse) (MOV10)/ Moloney leukemia virus 10-like 1, homolog (mouse) (MOV10L1)

MOV10 is a member of the UPF1-like superfamily1 of ATP-dependent RNA helicases and was first identified as a protein that prevents infection of mice by Moloney leukemia virus [224, 225]. It is a homolog of SDE3, a helicase for RNAi in *Arabidopsis*, and Armitage, a protein involved in RISC assembly and piRNA control of RNA viruses and endogenous retroelements in *Drosophila* [226, 227]. In humans, MOV10 associates with APOBEC3 proteins and components of RISC in SGs and PBs [156, 228]. Several groups examined the role of MOV10 in limiting HIV-1 replication but results were conflicted [229–233]. However, MOV10 strongly inhibits all human non-LTR retrotransposons in cell culture, consistent with its subcellular colocalization with L1 ORF1p in cytoplasmic granules, co-immunoprecipitation (co-IP) with the L1 RNP, and binding of L1 transcripts [232, 234–236]. Li et al. [237] showed that overexpression of MOV10 strongly reduced levels of exogenously expressed IAP and L1 RNAs at a post-transcriptional step, while inhibition of endogenous MOV10 increased RNA levels of transfected L1s. On the other hand, Lu et al. [238] found that MOV10 decreased IAP RT products but not IAP RNA or protein. The exact mode of MOV10 restriction remains uncertain. MOV10 binds mRNA surveillance protein UPF1 and promotes UPF1-induced nonsense-mediated decay, possibly by unwinding mRNA secondary structure and displacing proteins from 5' UTRs [235]. UPF1 itself binds both L1 ORF1p and ORF2p RNPs, and conceivably could recruit MOV10 to the L1 RNP. Paradoxically, however, while depletion of endogenous UPF1 increases L1 expression, cell culture retrotransposition is reduced [239]. Overexpression of UPF1 has no effect on retrotransposition [240].

MOV10L1, a MOV10 paralog, is expressed specifically in the mouse male germline and is required for both fertility and meiosis. Its RNA helicase activity is necessary for the proper biogenesis of pre-pachytene and pachytene piRNAs [241, 242] (reviewed in [243]). Loss of MOV10L in mice leads to depletion of MILI- and MIWI2-associated piRNAs, DNA demethylation in the testes, severe DNA damage in spermatids, and elevated expression of LINE-1 and IAP retrotransposons [244, 245].

### Zinc finger CCCH-type, antiviral 1 (ZC3HAV1/ZAP/PARP13)

ZAP is a member of the poly (ADP-ribose) polymerase (PARP) family of proteins. Human ZAP is a predominantly cytoplasmic protein that exists in two alternatively spliced isoforms, the shorter form being inducible by interferon (IFN) [246]. The longer isoform possesses a defective C-terminal PARP-like domain incapable of poly-ADP ribosylation. An N-terminal CCCH-type zinc finger domain binds and induces the degradation of transcripts from several positive and negative-strand RNA viruses, possibly by recruiting the RNA processing exosome and targeting viral RNA to cytoplasmic granules [247–249].

Both ZAP isoforms potently restrict cell culture insertion of non-LTR and mouse IAP retrotransposons through loss of retroelement RNA. ZAP closely colocalizes with L1 ORF1p and RNA in SGs, and binds the L1 RNP [236, 250]. While it is likely that ZAP recruits RNA degradation proteins to retrotransposon transcripts, inhibition of translation by ZAP has been reported for some viruses and cannot be excluded for L1s [251, 252]. Any roles for the exosome and SGs in ZAP-mediated retrotransposon restriction remain to be determined. Observations that both ZAP and MOV10 co-IP, overlap in cytoplasmic granules together with the L1 RNP, and promote loss of L1 RNA and proteins suggest the two proteins may act in the same pathway [250].

### Ribonuclease L (2',5'-oligoisoadenylate synthetase-dependent ribonuclease) (RNASEL)

RNaseL is an IFN-inducible endoribonuclease that binds and cleaves single-stranded regions of viral and cellular RNAs, and upon prolonged activation induces autophagy and apoptosis and the death of virus-containing cells. Viral double-strand (ds) RNAs activate oligoadenylate synthetase (OAS), which uses ATP to synthesize 2',5'-linked oligoadenylates (2-5As). 2-5A molecules bind latent RNASEL inducing its active dimer form (reviewed in [253]). RNASEL restricts retrotransposition of both IAP and L1 elements in cultured human cells, and causes loss of L1 RNA. Zhang et al. [254] hypothesized that RNASEL is activated by double-stranded regions existing within L1 RNA or that are formed by annealing of complementary transcripts generated by the sense and antisense promoters of the L1 5' UTR,

### Three prime repair exonuclease 1 (TREX1)

TREX1, the most abundant 3′–5′ DNA exonuclease in mammalian cells, targets reverse-transcribed retroviral cDNAs to prevent their accumulation in the cytosol. Paradoxically, TREX1 has also been identified as a cofactor for HIV-1 replication, and it has been proposed that HIV in part evades host innate immunity by exploiting TREX1 to clear its non-pre-integration complex cDNAs to levels unable to trigger cytosolic DNA receptors [255, 256]. Stetson et al. [257], showed that overexpression of TREX1 dramatically reduced retrotransposition of L1 and IAP elements in cell culture, and that ssDNA fragments from endogenous retroelements, including LINE-1s, SINEs and ERVs, accumulate in heart cells of *Trex1* KO mice, demonstrating that TREX1 metabolizes reverse transcribed cDNA.

### Others

Other cellular proteins strongly inhibit retrotransposition, mostly by unknown mechanisms. For example, melatonin, the hormonal regulator of circadian rhythms and sleep, and its MT1 receptor suppress L1 expression in an in vivo cancer model and dramatically decrease retrotransposition in cultured cells [258]. Poly-A binding protein C1 (PABPC1) is important for L1 RNP formation, and perturbing its levels alters cell culture retrotransposition and subcellular localization of ORF1p [259]. An affinity capture screen of factors that bind the internal ribosome entry site (IRES) of mouse L1 RNA revealed HNRNPL and nucleolin, whose depletion, respectively, increased and decreased mouse L1 cell culture retrotransposition 10-fold [260, 261]. Evidence suggested that while nucleolin functions as an IRES-dependent trans-acting factor for mouse ORF2 translation, HNRNPL behaves like a host restriction factor by decreasing levels of L1 RNA and protein. In separate studies, human HNRNPL bound the L1 RNP and strongly reduced cell culture retrotransposition [56, 236, 240].

TEX19.1 is a mammalian-specific protein of unknown function whose expression is limited to germ and pluripotent stem cells, and the placenta [262]. Mouse TEX19.1 is important for normal placenta development and spermatogenesis. It also represses expression of transposable elements, including MMERVK10C LTR elements in the male germline and LINE-1 in embryonic stem cells (ESCs) and hypomethylated trophectoderm-derived cells of the placenta [263–265]. Although the mouse *Tex19.1* KO phenotype resembles those of *Miwi2* and *Mili* mutants, there are indications that TEX19.1 protein may inhibit retroelements at a post-transcriptional step, distinct from the piRNA pathway [263].

MARF1 is an essential regulator of mouse oogenesis, and loss of function causes infertility in females only. LINE-1 and IAP retrotransposon expression is upregulated in mutant Marf1 oocytes coincident with an increase in dsDNA breaks. While its mechanism of inhibition is unknown, structural similarities have been noted between MARF sequence and RNase-like and RNA binding-motifs of PIWI and TDRD5/7 proteins, respectively [266, 267]. Limkain B, the human orthologue of MARF1, is a component of P-bodies [268].

Macroautophagy traps cellular components in double-walled vesicles called autophagosomes and delivers them to lysosomes for degradation. Autophagy also plays a role in the metabolism of Alu and L1 transcripts, which colocalize and copurify with autophagosomes. Knockdown of autophagy receptor proteins increased Alu and L1 cell culture retrotransposition, and qPCR analyses showed LINE-1 insertions to increase in mice lacking the autophagy regulatory protein Beclin1 (BECN1/ATG6) [52]. Autophagic control of retrotransposition is a strategy also conserved in *Saccharomyces cerevisiae*, which targets Ty1 VLPs to autophagosomes via interaction with Atg19p [269].

Adenosine deaminase acting on RNA (ADAR) proteins bind dsRNAs and convert adenosines to inosines. Both antiviral and proviral roles have been reported for ADARs (reviewed in [270]). In humans A-I RNA editing occurs primarily in Alus present in the non-coding regions of pre-mRNA transcripts [271–274]. Alus with inverted orientations and proximal to each other, and there are many in the human genome, form dsRNA stem loop structures that are preferred templates for ADAR editing. Editing of Alus has been linked with alternative splicing, gene silencing, and altered RNA transport (reviewed in [275–277]). While roles for ADAR editing in the evolution of Alu subfamilies and the suppression of their retrotransposition by mutation has not yet been determined, it is logical to assume they exist.

Yeast Two Hybrid assays and recent affinity-capture and co-IP experiments have identified many other predominantly RNA-binding proteins that bind and colocalize with L1 RNP complexes. Some of these proteins strongly repress cell culture retrotransposition when overexpressed and are obvious candidates for future investigation [51, 56, 236, 239]. CSDA, DDX39, HNRNPA1, HNRNPU, MX2, PURA, SRSF1, and YB1 form a partial list. Roles for many of these proteins in viral replication are known.

## The nuclear option

Most of the restriction factors described so far function largely in the cytoplasm, limiting retrotransposition by post-transcriptional mechanisms. Other factors function in the nucleus, suppressing transcription at the first step of retrotransposition, or interfering with DNA integration at the last (reviewed in [278, 279]).

In plants much crosstalk exists between DNA methylation, histone modification, and RNA interference, each of which has been implicated in transcriptional silencing of retrotransposons [280, 281]. Our understanding of their united effects in mammals is less developed and derives mainly from studies in mouse ESCs and embryos and extensive work on the regulation of ERVs. Repression of IAP retrotransposons in early mouse embryogenesis is maintained primarily by histone methylation, but in post-mitotic germ and other differentiated cells DNA methylation assumes importance [222, 282–284]. SINEs, LINEs and SVAs typically bear histone H3 methylated at Lys9 (H3K9me2/me3) repressed chromatin marks, and H3K9 methyltransferases EHMT2/G9A and SUV39H have been implicated in their repression [285–289] (although Dong et al. [290] failed to detect increased expression of LINE-1s in a *G9A*^−/−^ cell line despite their hypomethylation). Inhibition of SUV39H In human cells reduces H3K9 histone trimethylation and stimulates recruitment of polymerase III together with increased expression of some subfamilies of Alu [291]. Loss of ESET/SETDB1 methyltransferase in mouse PGCs is marked by a decrease of H3K9me3 and H3K27me3 marks on LTRs and LINE-1s, with widespread transcriptional derepression of ERVs but not L1s [284, 292]. Other repressive histone marks may be enriched on non-LTR retrotransposons, although the predominant mark may vary with cell type and species, and discrepancies between study results exist [285, 286, 293–295]. Fadloun et al. [296], for example, found that repression of L1s during preimplantation follows loss of active chromatin marks such as H3K4me3 rather than gain of repressed H3K9me3 marks.

Additional chromatin-associated proteins have been implicated in repression of non-LTR retrotransposons. Loss of histone chaperone chromatin assembly factor 1 (CAF-1) leads to significant up-regulation of L1s, B2 SINES, and IAPs in morula-stage mouse embryos, together with increased histone H2AX phosphorylation and developmental arrest. Treatment with RT inhibitors rescues some of these embryos and so implicates retrotransposon activation in their arrest [297]. Mouse embryonic fibroblasts (MEFs) deficient for all retinoblastoma susceptibility protein family members show upregulation of L1 expression and diminished HDAC1, HDAC2 and NuRD (nucleosomal and remodeling deacetylase) corepressor complex recruitment with consequent epigenetic changes at the L1 promoter [112, 298]. Retrotransposition has salted the human genome with p53 transcription factor binding sites present in the L1 5' UTR, with potentially significant effects on the expression of neighboring genes [110]. Functional p53 represses DNA damage-induced SINE transcription [299]. Loss of p53 increases activity of *Drosophila* non-LTR retrotransposons, and a human L1 introduced into tp53-mutant zebrafish showed increased retrotransposition and loss of H3K9me3 marks on the 5'UTR. Elevated LINE-1 expression is also a feature of p53 mutant cancer cell lines [300].

KRAB-associated protein 1 (KAP1/TRIM28) is a transcriptional corepressor essential for normal development and cell differentiation. KAP1 mediates the recruitment of chromatin remodeling complexes to DNA by binding Krüppel-associated box domain-containing zinc finger proteins (KRAB-ZFPs) and other DNA-binding proteins (reviewed in [301, 302]). Roles for KAP1 and its KRAB-ZFPs in the control of both exogenous and endogenous retroviruses of mice are well established [303–307] (reviewed in [31, 308]). In mouse ESCs, silencing of many ERV elements is maintained by SETDB1-mediated H3K9me3 methylation, and KAP1 is required for their repression [309–312]. In human ESCs, KAP1 is recruited to older L1PA6 to L1PA3 subfamilies, but is largely absent from young human-specific L1Hs elements. Its binding is associated with H3K9me3 enrichment and its depletion with expression of these older elements [313]. Some non-LTR retrotransposons are bound by species-specific KRAB-ZFPs, including mouse ZFP819, which inactivates LINES and SINES [314], mouse GM6871, which weakly suppresses two relatively young but retrotransposition-incompetent mouse L1 subfamilies (L1MdF2 and L1MdF3) [313], and ZNF91 and ZNF93, for which there is evolutionary evidence for suppression of now inactive human SVA and L1 subfamilies [315] (reviewed in [316]). ZBTB16/PLZF, a regulator of cell growth and differentiation, also binds L1 DNA, altering local chromatin acetylation and methylation, and repressing L1 expression in germ and progenitor cells and retrotransposition in the cell culture assay [317].

Methylation regulation of retrotransposons is complex and controlled by interacting factors whose activity has been linked to mammalian germline development. Half the CpGs in the human genome reside in repeats, 25 % of them in Alus and 12 % in LINEs [318, 319]. While most CpG islands in gene promoters are undermethylated if the genes are expressed, an island in the L1 5' UTR is typically heavily methylated in somatic cells and L1 expression is suppressed [320–323]. Indeed, it has been proposed that DNA methylation of CpGs evolved primarily as a host defense mechanism against TEs [324]. Garcia-Perez et al. [325] made the interesting observation that in human embryonic carcinoma-derived cell lines an L1 reporter was strongly silenced by methylation during or shortly after retrotransposition and could be desilenced with histone deacetylase inhibitors; a control reporter gene inserted alone into the genome was not. This suggested that epigenetic silencing specifically targets the TPRT event. Significantly, methylation and other repressive chromatin marks may spread beyond LTR and non-LTR insertions into flanking DNA, and effects on the expression of nearby genes are possible [326–330].

In adult mice, IAP elements are heavily methylated in mature eggs and sperm, but L1s are undermethylated in eggs compared with sperm and somatic cells [331, 332]. Successive waves of demethylation occur in the developing mouse embryo. The first wave is shortly after fertilization until the morula stage and involves LINES, SINES and LTRs. Demethylation occurs again around E8.5 in post-implantation primordial germ cells that are entering the hindgut endoderm, and continues through to E12.5 to E13.5 when PGCs have colonized the genital ridges. LINE-1 methylation is largely erased in PGCs, but IAP CpGs remain more resistant (summarized in [181, 333–337]). Some retrotransposons evade the remethylation that occurs post-E13.5 and are subject to piRNA pathway-mediated methylation from E16.5 [202, 338]. However, substantial demethylation of L1s is not always mirrored by transcriptional activation [337].

DNMT1, the most abundant DNA methyltransferase (MTase) in mammals, preferentially methylates hemimethylated DNA (maintenance methylation); DNMT3 MTases are more involved in *de novo* methylation of unmethylated CpGs. However, the three MTases function cooperatively. Both IAPs and L1s are demethylated in *Dnmt1*^−/−^ mouse ES cells [339–344]. B1 SINEs are methylated by DNMT3A, and IAP and LINE-1 elements are methylated by both DNMT3A and DNMT3B. DNMT3L regulatory factor lacks catalytic activity, but recruits DNMT3A and DNMT3B to their targets. In nondividing prospermatogonia, DNMT3L functions mainly in establishing methylation of retrotransposons, including L1s and IAPs. Deletion of *Dnmt3a* or *Dnmt3l* results in uncontrolled transposon expression in the mouse male germline with spermatogenesis failure and sterility [342, 345]. Zamudio et al. [222] found that DNA methylation is dispensable for TE silencing prior to male germ cell meiosis. With the onset of meiosis and programmed loss of chromatin repression, L1s were activated in *Dnmt3l*^−/−^ mice, accompanied by precocious loss of H3K9me2 marks and gain of H3K4me3 marks and SPO11-induced dsDNA breaks in TEs. However, no attendant increase in TE genomic copy number was detected by qPCR.

In addition to MTases, several cofactors are involved in DNA methylation and suppression of TEs. Lymphoid-specific helicase (LSH/HELLS) belongs to the SNF2 helicase family of chromatin remodeling proteins and may recruit DNMT3B to chromatin [346]. LSH is essential for normal embryonic development, and its loss in mouse embryos or the female germline is accompanied by DNA demethylation, altered histone acetylation, abnormal heterochromatinization, and hypomethyation of pericentromeric satellite repeats and IAP elements [347, 348]. In the absence of LSH, IAPs are upregulated, but LINE-1 sequences remain repressed despite being hypomethylated [349]. UHRF1 (also known as NP95 in mice and ICBP90 in humans) is another cofactor that recruits DNMT1 to hemimethylated CpGs. Its loss leads to hypomethylation and upregulation of IAPs, L1s, and SINEs in mouse embryos [350, 351]. In the mouse male germ line, MORC1, a member of the Microrchidia (Morc) family of GHKL ATPases, regulates repression of various families of retrotransposons, including L1s. Although KO mice suffer germ cell loss and infertility similar to animals defective for piRNA proteins, MORC1 appears to act independently of the piRNA pathway and may facilitate DNA methylation of TEs [352]. Morc family homologs in *Arabidopsis* also repress transposons [353].

DNMT3A and DNMT3B also mediate methylation-independent gene repression through their association with heterochromatin protein 1 (HP1), methyl-CpG-binding proteins (MeCP), and histone MTase activity [354]. MECP2 binds methylated CpG dinucleotides and forms complexes with DNA MTases or histone deacetylases. In cell culture, MECP2 binds the L1 5' UTR to limit expression and retrotransposition [355]. Retrotransposon expression is increased, and, as shown by qPCR, L1 copy numbers are elevated in brains of *Mecp2* KO mice as well as in patients with Rett syndrome, a rare neurodevelopmental disorder caused by mutations in *MECP2* [356, 357].

The piRNA pathway not only degrades RNAs post-transcriptionally but may induce gene silencing by promoting DNA and histone methylation (reviewed in [358]). PIWI proteins play important roles in *de novo* DNA methylation. The fact that *de novo* CpG DNA methylation and transcriptional silencing of transposable elements is reduced during fetal spermatogenesis of *Mili* and *Miwi2* KO mice suggests that piRNA RISC recruits methylation proteins to TE loci, including the L1 5' UTR [200–203, 359]. The piRNA pathway is also important in mouse germ cells for deposition of H3K9me3 marks on young active L1s [329], as well as on TEs in flies [360, 361].

The enzymes that catalyze DNA and histone methylation are well characterized; those that remove methyl-groups much less so. Deficiency of maternal LSD1/KDM1A histone H3 mono- and dimethyl K4 demethylase during early mouse development leads to desilencing of muERV-L/MERVL elements and LINE-1s, and an increase in L1 ORF1p expression that is most obvious in cell nuclei [362, 363]. TET1 (ten-eleven translocation) enzyme oxidizes 5mC to 5-hydroxymethylcytosine (5hmC), an intermediate for the removal of 5mC [364]. 5hmC is enriched at the promoters of L1s in murine ESCs [365]. Recently the notion that DNA methylation in mammals occurs only as 5-methylcytosines was challenged with the discovery of N6-methyladenine modification in mouse ES cells. Knockout of the demethylase gene *Alkbh1* caused increased N6-mA deposition on the 5' ends of young but not old L1s that correlated with an increase in other repressive marks on the L1s and transcriptional silencing of nearby genes and enhancers [366]. Discoveries such as these add new layers of complexity to the regulation of retrotransposons by DNA methylation

DNA repair proteins have been implicated in modulation of retrotransposition, although their roles in TPRT are unclear. Endonuclease-independent retrotransposition is strongly elevated in Chinese hamster ovary cells lacking non-homologous end-joining (NHEJ) repair proteins [68, 72]. Chicken DT40 cell lines defective for NHEJ genes, including *DCLRE1C* (artemis), *LIG4*, and *XRCC6/KU70*, show restricted retrotransposition of transfected human L1 and zebrafish ZfL2-2 LINE2 elements [367]. The ERCC1/XPF heterodimer is involved in nucleotide excision, recombination, and inter-strand crosslink repair, and limits non-LTR retrotransposition in cell culture [368]. Ataxia telangiectasia mutated (ATM), a serine/threonine protein kinase activated by dsDNA breaks, has been linked with retrotransposition, although results are contradictory. Cell lines mutated for *ATM*, or with ATM protein levels reduced by expression of human papillomavirus E6 oncoprotein, had attenuated L1 activity, implying a supportive role for ATM in retrotransposition [369, 370]. On the other hand, ATM-deficient neuronal precursor cells and the brains of *Atm* KO mice showed elevated activity of an L1-mEGFPI reporter transgene (Fig. 3), and ataxia telangiectasia patients had increased L1 copy numbers as detected by PCR [371]. Thomas et al. [372] suggested that use of G418 antibiotic to select the L1-*mneoI* reporter construct used by Gasior et al. [369] may have caused cell toxicity and affected results (although, GFP-induced cytotoxicity has also been reported [373]).

Despite the numerous cellular proteins implicated in the control of mammalian non-LTR retrotransposon integration, beyond first strand synthesis, no comprehensive model of integrant resolution and repair exists. This failure has been in part due to the lack of an effective *in vitro* assay that recapitulates later steps of the TPRT reaction. Such assays have been instrumental in detailing the mechanisms of genome insertion by bacterial and yeast Group II introns and insect R1 and R2 retrotransposons [374, 375]. Although Cost et al. [376] reconstituted the initial stages of L1 element transposition *in vitro*, the field has failed to take up the challenge to refine this assay and apply it to mechanistic investigations.

## An arms race

It has been proposed that cells are engaged in a genetic “arms race” with infecting retroviruses and endogenous retrotransposons, and must constantly evolve new strategies to fight infection or transposition. This places selective pressure on the parasitic element, which contrives to evolve measures to evade repression, which in turn may be countered by new changes within a host restriction factor [377]. One signature of the struggle between host and pathogen is positive (diversifying) selection for alleles that confer fitness benefit. For examples, the C-terminal PARP-like domain of ZAP-L displays recurrent positive selection and enhanced ZAP-mediated anti-viral and anti-retrotransposon activites [250, 378]. APOBEC3A has undergone diversifying selection in response to a changing repertoire of viral pathogens, while maintaining the ability to inhibit L1s through 40 million years of primate evolution [379]. A recent study by Jacobs et al. [315] provided two examples of dynamic coevolution between KRAB-ZFPs and their retrotransposon targets. Eight to 12 million years ago, a series of structural modifications enabled ZNF91 to bind and repress SVA elements. In turn, until about 12.5 million years ago, ZNF93 suppressed early primate L1s until there arose the younger L1PA3 subfamily that lacked the ZNF93 target sequence. It has been proposed that KRAB-ZFP genes reflect a classic arms race between retroelements and their hosts, with ZFP repressors evolving novel DNA binding specificities that target retrotransposon subfamilies as they became newly active in the genome [380]. Indeed, of 18 KRAB-ZFPs tested, 16 bound specific classes of endogenous retroelements [381]. Moreover, the predicted ages of the ZFPs and the retroelements they bound were correlated with only two exceptions, ZNF33A, which primarily bound SVAs, and ZNF382, which associated with younger L1Hs elements.

LINE-1 type Transposase Domain-containing 1 (L1TD1/ECAT11), the sole known example of functional domestication of LINE-1-derived protein sequence, contains two ORF1-like domains. L1TD1 is associated with self-renewal and the maintenance of pluripotency in embryonic stem cell culture (although not in KO mice) [382, 383]. Its loss or pseudogenization in multiple mammalian lineages, together with evidence for diversifying selection, prompted McLaughlin et al. [384] to propose that L1TD1 originally evolved as a host restriction factor against retrotransposons that was later coopted as a pluripotency factor. Like L1 ORF1p, endogenous L1TD1 is detected in PBs [382], although any effect on L1 activity is so far unknown. Conceivably, expression of L1TD1 protein might exert a dominant-negative effect on ORF1p function.

There is some evidence for positive selection within and in the near vicinity of L1s. Using genome-wide analyses, Kuhn et al. [385] detected extended haplotype homozygosity around some L1 insertions with evidence for recent positive selection; this predicts potential phenotypic effects of the L1s, although, no supporting functional studies were attempted. Since the mammalian radiation, a single lineage of L1s has been active in both mice and humans, each subfamily losing activity due to mutations, to be then supplanted by the next, until today there remains one active subfamily in humans (L1PA1) and three in mice (A, T_F_, and G_F_) [386, 387]. Positive selection is evident in the coiled-coil domain of human L1 ORF1p; coiled-coils mediate protein-protein interactions [19, 20, 388]. Although the coiled-coil domain of mouse ORF1 fails to show positive selection, there has been considerable structural instability in this region. It has been suggested that the diversity of 5' UTRs and novel ORF1 sequence variants that distinguish mouse L1 subfamilies arose from recombination and may reflect an evolutionary drive for the L1 to adapt to cellular host factors [46, 47, 389].

Some lentiviruses have evolved small accessory proteins that both modify cellular functions and mute the cell’s antiviral response. Vif and Vpx, for example, target APOBEC3G and SAMHD1 for ubiquitination and degradation, and BST-2 is neutralized by HIV-1 Vpu, SIV Nef, and HIV-2 Env (reviewed in [390–392]). It is therefore reasonable to consider that by disrupting host restriction factors, HIV infection might stimulate retrotransposition, Indeed, this effect was observed [393], and expression of Vif or Vpr was necessary for maximal induction of HIV-infected Jurkat cell culture retrotransposition. An increase in L1 and Alu DNA copy numbers was also detected by qPCR of DNA from infected CD4^+^ T cells, but could not be confirmed to represent new insertions [393]. Vpr is a multifunctional accessory protein that regulates nuclear import of the HIV-1 preinitiation complex; it is not known if it targets a host restriction factor [394]. Recombinant Vpr protein added to cell culture increased tagged L1 retrotransposition, and when injected into transgenic mice caused an increase in genomic L1 copy number as determined by qPCR [395, 396].

At this point, a note of caution may be in order. Commencing with investigations of retrotransposition in brain tissue samples [356, 371, 397, 398], the use of sensitive qPCR strategies to assess variation in the copy number of L1 genomic insertions is becoming *de rigeur* in the field. This trend is likely to increase with the development of more sensitive digital droplet PCR protocols [399]. Apparent changes in retrotransposon copy number are never confirmed by downstream genome sequencing to detect new insertions. Previously, I proposed a possible source of bias for such PCR-based studies [400]. Cellular conditions that stimulate expression of L1s or HERVs, and therefore their encoded reverse transcriptases, might also induce promiscuous reverse transcription of retrotransposon RNAs not engaged in TPRT at the site of chromatin integration. The cDNAs so generated would be amenable to qPCR amplification, biasing upwards estimates of genomic L1 copy numbers. Although an unverified concern, recent studies suggest that it is not an unreasonable one. For example, elevated levels of Alu- and LINE-1-containing hybrid RNA/DNA molecules have been detected in cancer cell lines and are lost upon treatment with RT inhibitor [401]. cDNA complementary to infecting viruses and cellular mRNA is generated independent of genomic integration in the presence of LINE-1 ORF2p [402, 403]. Protocols that isolate only high molecular weight DNA (such as gel purification) and that apply RNase H (to degrade RNA/DNA substrates) and ssDNA nucleases prior to qPCR could remove contaminating molecules that might confound data interpretation.

Since the 1980s, extrachomosomal small polydispersed circular (spc) DNAs containing retrotransposon sequences, including SINEs and L1s, have been reported in cells. Recombination, replicon misfiring, modified TPRT, and reverse transcription models have been proposed to explain these extrachromosomal DNAs [404–407]. Their copy numbers are elevated in cancer cells and associated with genome instability [408, 409]. Still a poorly studied class of mammalian copy number variants, spcDNAs could conceivably be an additional off-target source of amplicons for some PCR-based analyses of genomic retrotransposon insertions. That said, standardized PCR protocols that reliably detect retrotransposon insertion copy numbers would be a boon to the field.

RT-PCR assessment of retrotransposon expression is also prone to misinterpretation. One must be confident that only L1 RNAs transcribed from the 5' UTR promoter are amplified. Amplification of unrelated mRNAs that by chance contain retrotransposon PCR target sequence will bias results. When designing primer pairs, *in silico* analyses of potential off-target binding sites in mRNAs should be performed. Studies published to date do not report such analyses, and frequently qPCR results are not validated by other techniques measuring L1 expression. As well as being part of longer mRNA transcripts, L1s generate full length, spliced and prematurely polyadenylated products from their sense promoters. Northern blotting protocols that provide information about the 5' ends and length of L1 transcripts should be the gold standard of analysis (these issues are discussed in [410]).

## When the defenses fail

If we think of retrotransposons as genetic parasites, it makes sense they should have evolved to be active in the germline and transmit to future generations, but remain inactive in somatic cells and not risk harming the host. This notion was dispelled by F. Gage and colleagues at the Salk Institute who showed that L1 retrotransposition occurs in neuronal precursor cells, especially in the hippocampus [106], and by the Boeke and Kazazian labs who showed retrotransposition in early mouse and human development, implying that each of us is a mosaic of somatic genomes [411–413]. Other papers using HT genome sequencing have since concurred that there is endogenous somatic L1 retrotransposition in neural precursors and the adult brain, although estimates of insertion frequency differ by more than an order of magnitude [170, 371, 414–416] (reviewed in [372, 417–419]. The Faulkner group [170], using retrotransposon-capture sequencing (RC-seq) of single cells, estimated high rates of L1 retrotransposition in the hippocampus (averaging 13.7 insertions per neuron) and cerebral cortex (16.3 per neuron). The Walsh and Park groups [415, 416] reported a much lower average of <0.6 of an insertion per neuron using L1Hs insertion profiling (L1-IP). Following somatic transfer and expansion in oocytes of six post-mitotic nuclei of mouse MT (middle temporal visual area) neurons, Hazen et al. [420] found an average of 1.3 new insertions per neuron. Recently, Evrony et al. [421] reanalyzed the Faulkner group data, criticized aspects of its bioinformatic and validation approaches, and concluded a revised estimate of 0.2 of an event per neuron. It has been suggested, however, that this reanalysis made both inappropriate use of a post-filtered dataset and erroneous assumptions in concluding chimeric artifacts in the Upton et al. [170] PCR-validations (G. Faulkner, pers. comm.). Nevertheless, even the low estimate of 0.2 of an insertion per neuron predicts 20 billion unique insertion events in a human brain. There is evidence based on tagged engineered L1 assays that retrotransposition is not limited to neuronal precursor cells but can take place in non-dividing mature neuronal cells as well (J. Garcia-Perez, pers. comm.). It has been proposed that retrotransposition contributes to neuronal plasticity (reviewed in [422]), although brain tumors seem as likely a consequence. However, to date no *de novo* L1 insertions have been detected in glioblastoma or medulloblastoma brain cancers [423–425].

Why suppression of non-LTR retrotransposons is perturbed in some but not other cell types is unclear, but has implications for development and disease. L1 promoter hypomethylaton, elevated L1 expression, and cell culture retrotransposition have been demonstrated in human iPSC and ESC lines [426–433] (reviewed in [434, 435]). Interestingly, iPSCs from non-human primates support greater cell culture retrotransposition than human iPSCs, correlating with lower levels of APOBEC3B and PIWIL2 proteins in the former, and the significantly larger pool of chimpanzee-specific L1 elements [432]. Recently, retrotransposition of endogenous L1, Alu and SVA elements has been shown to occur during reprogramming of human iPSCs and in pluripotent stem cell culture [436, 437].

There is limited data on endogenous retrotransposition in normal somatic adult tissues other than the brain, except for the finding of a single potential somatic insertion in hepatocytes [438] and small numbers of insertions detected in DNA of esophagus, stomach and colon [439–441]: at least some of these insertions may have occurred during early embryogenesis. On the other hand, many *de novo* insertions have been detected during HT sequencing analyses of bulk cancer tissues. In 2010, Iskow et al. [423] first reported tumor-only L1 insertions in lung cancer, and subsequent studies have made it clear that somatic retroelement insertions are detectable at varying frequencies in a subset of tumors, especially those of epithelial origin [424, 425, 438–444]. These insertions may have sequence characteristics that differ from typical germline insertions, such as a higher degree of 5’ truncation and more frequent insertions independent of L1-encoded endonuclease cleavage [445].

A role for retrotransposition in the etiology of cancer remains an open question, however (reviewed in [446–448]). To date there have been a few “smoking gun” examples of tumor-specific L1 insertions presumed to have led to cancer. In 1992 Miki et al. [449] showed that an L1 had inserted into an exon of the APC tumor suppressor gene in a colon cancer, but that the insertion was undetectable in normal colon of the affected individual. Over 20 years later, tumor-specific L1 insertions were found in the Suppression of Tumorigenicity18 (ST18) gene of a hepatocarcinoma [438], in an exon of the PTEN gene in endometrial cancer [425], and in the APC gene of a colorectal cancer [450]. Recent work indicates that many insertion events detected in tumors were already present in precancerous lesions, and were perhaps present in the somatic cells that gave rise to the tumors. Some insertions were detected only in metastases and not in the primary tumors, suggesting late cancer-specific events [440, 444]. We do not, however, know the background levels of retrotransposition in normal somatic cells and if these levels are sufficiently frequent to be of significance for cancer progression or somatic disease. We do not know if retrotransposition may drive cancer, or cancer accelerates retrotransposition. Interestingly, there is evidence for the misregulation of AID and APOBEC3 in some cancers leading to increased mutation and perhaps contributing to clonal evolution and tumor progression [451, 452] (summarized in [453, 454]). Increased cytosine deaminase activity could tamp-down retrotransposition in tumors; it might also be an induced response to increased retroelement expression.

Expression of L1s and their ORF1 or ORF2 proteins is altered in various tumor types compared with their normal tissues, phenomena that may prove useful as diagnostic markers of cancer progression [57, 60, 439, 455–458]. Hypomethylation of L1 DNA has been observed in many cancers and is associated with increased L1 expression [459, 460]. One might therefore expect increased expression to mean increased retrotransposition. However, no study to date can conclude that endogenous retrotransposition frequency is specifically elevated in cancer since only tissues in bulk have been sequenced. In non-tumor tissue, an individual *de novo* insertion will be present in only a small subset of cells among the large total population of cells sampled, and may exist in too low a copy number to be detected by standard amplification methods. An insertion initially present within a normal cell is more easily detected once that cell clonally expands as a tumor, which, upon sampling, sequencing and PCR validation, would falsely appear to possess a tumor-only event (discussed in [400]). Single-cell sequencing protocols should provide true estimates of the rates of retrotransposition in tumor versus normal somatic cells.

Misregulated expression of retrotransposons can damage the genome. The endonuclease activity of the L1 ORF2 protein generates a dsDNA break that recruits repair proteins to the retrotransposon insertion site. This is a normal part of TPRT. However, transient transfection of L1s in cell culture induces DNA breaks far in excess of what would be expected for TPRT alone [369]. DNA damage caused by overexpression of ORF2p may induce genotoxic stress and cellular apoptosis [69, 369, 461, 462]. Elevated ORF2 endonuclease and RT activities in mice have also been linked with increased meiotic prophase I defects and fetal oocyte attrition, a mysterious process that involves loss of a majority of oocytes prior to birth [463]. The fact that treating mice with the nucleoside analog AZT blocks oocyte attrition, suggests that RT inhibitors might be applied to suppress retrotransposons and perhaps extend the female reproductive lifespan [464]. One might also wonder if epigenetic misregulation or loss of a restriction factor causing elevated retrotransposon activity could trigger diminished fertility or even spontaneous abortions in humans, topics worthy of further investigation.

Several reviews have linked the aging process with progressive changes in chromatin architecture and increased expression of retrotransposons [465–469]. Increased mobilization of gypsy and non-LTR R1 and R2 retrotransposons in the aging fly brain is accompanied by neural and cognitive decline [470]. Senescence of fibroblasts and aging mouse tissues are marked by progressive epigenetic reorganization, depression of retrotransposons, and increased insertions at late-stage senescence as determined by qPCR [471, 472]. Longevity-linked protein Sirtuin-6 (SIRT6) represses L1s by binding their 5' UTRs, and promotes heterochromatinization through mono-ADP ribosylation of KAP1. SIRT6 vacates L1 loci in senescent cells and brain tissues of aging mice, with an accompanying increase in L1 transcription and PCR-detected insertions [473]. However, while senescence may foster retrotransposition, the notion that retrotransposition hastens aging, as with the notion that it significantly promotes cancer, remains speculative. Elevated ORF2 endonuclease expression and TPRT cause DNA damage and genomic lesions, and certainly DNA damage increases with age. However, a direct connection between these phenomena is unclear.

Most links between retrotransposons and disease involve endogenous retroviruses. Altered HERV expression occurs in SLE, Sjogren’s syndrome, multiple sclerosis, schizophrenia, psoriasis, Creutzfeldt-Jakob disease, amyotrophic lateral sclerosis, and various cancer conditions, although the specific HERV loci that contribute to the transcriptional misregulation observed have been only partially documented and a causative role for these sequences in disease is largely speculative [474–478] (reviewed in [479–484]). Among the more convincing studies, elevated expression of HERV-K transcripts in cortical and spinal neurons of ALS patients is supported by evidence of neurotoxic effects of the HERV-K env protein in a mouse model [485]. Syncytin1 protein, which derives from the envelope gene of the HERV-W ERVWE-1 locus, has essential functions during placental development and is upregulated in multiple sclerosis. Its expression causes cytotoxicity of astrocytes in vitro and oligodendrocyte loss and demyelination in transgenic mice (reviewed in [486]).

Links between misregulated non-LTR retrotransposon expression and disease are fewer. Expression of Alus is altered in certain neurodegenerative conditions, including Creutzfeldt-Jakob and Alzheimer diseases [487]. DICER deficiency in geographic atrophy, a form of age-related macular degeneration, induces accumulation of Alu RNA, which in turn activates the NLRP3 inflammasome complex and downstream caspases leading to retinal pigment epithelial cell death [488, 489]. In a study that proved Sjögren’s syndrome autoantigen RO60/SS-A binds Alu RNAs, transfection of the bound Alu motif into peripheral blood cells stimulated proinflammatory cytokine secretion, while IFN-α treatment of *RO60*-null lymphocyte cells activated Alu transcription [490]. Yu et al. [491] further showed that increased L1 expression in human fibrosarcoma cells and in testes and MEFs of *Mov10l* KO mice is marked by induction of IFN-β and IFN-stimulated genes (although, not unexpectedly, elevated IFN levels also inhibit L1 cell culture retrotransposition, perhaps by inducing restriction factors [250, 491]). Such results link retrotransposon RNA metabolism with the immune response.

Aicardi–Goutières syndrome is a rare inflammatory disorder with no known cure. Within the first year of life, patients usually experience severe brain dysfunction and neurological damage that clinically mimics *in-utero* viral infection. AGS is characterized by increased IFN and IFN-stimulated gene expression. The condition has been associated with mutations in seven genes, including *SAMHD1, TREX1, RNASEH2A, RNASEH2B, RNASEH2C, ADAR1, and IFIH1* [492, 493]. *TREX1* mutations are also implicated in other autoimmune conditions, including systemic lupus erythematous, chilblain lupus, and retinal vasculopathy with cerebral leukodystrophy [494, 495]. Significantly, AGS-associated genes are involved in DNA or RNA metabolism and are components of the intrinsic immune response against exogenous retroviruses or endogenous retroelements. It has been proposed that a defect in an AGS-related gene prevents the cell from efficiently “clearing” endogenously-produced nucleic acids, causing their accumulation in the cytoplasm where they bind pattern recognition receptors and trigger an innate type I interferon response. One possibility is that these self-nucleic acids derive from the RNA or reverse transcribed cDNA of retroelements, although this remains untested [257, 482, 496]. Significantly, Stetson et al. [257] found that ssDNA fragments from endogenous retroelements accumulate in heart cells of *Trex1*-null mice, possibly contributing to their characteristic inflammatory myocarditis and death [497] (although a more recent study failed to detect increased ERV expression in Trex1^−/−^ mouse dendritic and macrophage cells [498]). Significantly, a combination of nucleoside RT inhibitors expected to inhibit both retroviruses and L1 retrotransposons attenuated the autoimmune myocarditis [499]. Encouraged by such insights, an early stage RT inhibitor clinical trial for children with AGS has begun in Paris (https://clinicaltrials.gov/ct2/show/NCT02363452).

## Aftermath

Retrotransposons pose an ongoing threat to the human genome. In the past six million years, 1174 fixed LINE-1s, 5530 Alus, and 864 SVAs have accumulated in hominins [500]. In addition, each of us possesses rare and potentially active insertions. Extrapolating, the world’s population of 7 billion may therefore harbor millions of active unique or low allele frequency human-specific non-LTR retrotransposons [66]. While low levels of retrotransposition and other mutations in the germline maintain genetic variation, high levels may jeopardize viability of the cell. Moreover, if, as our field is coming to believe, retrotransposition in somatic cells is much greater than in the germline, it could be a significant source of cell-to-cell variation. Retrotransposon activity may play key roles not only in Mendelian disorders, but in multifactorial disorders and cancers as well. It is therefore important to better understand how the cell limits retrotransposons and why its defenses occasionally falter.

Retotransposition is elevated in early embryogenesis, stem cells, neuronal progenitor cells, and perhaps some cancers. Misregulation of RT expression is detected in some disease conditions, and RT inhibition has been reported to inhibit cell proliferation and tumor growth, alter embryogenesis, and promote cell differentiation [401, 501–503] (see reviews [504, 505]). These observations deserve greater investigation. While not reviewed here, a rich literature also demonstrates that environmental stress, including carcinogens, heat shock, heavy metals, addictive stimulant drugs, ionizing radiation and steroids, induce mammalian non-LTR retrotransposons, with consequences for humans that remain to be determined (reviewed in [506–512]).

The repression of retrotransposons occurs on two main fronts: post-transcriptionally and mostly in the cytoplasm, and in the nucleus where histone modification and DNA methylation limit transcription (Table 1). The epigenetic derepression that occurs during early embryogenesis and germ cell development in mammals leaves the cell vulnerable to retrotransposition. Thus, piRNA pathway proteins have evolved to be important guardians of germline genomes. Some cytoplasmic restriction factors are part of the intrinsic immune system of the cell, although their battle strategies may sometimes be unclear. APOBEC3 proteins, for example, may sequester non-LTR retrotransposon RNPs in cytoplasmic aggregates or may deaminate their cDNAs during TPRT. While SAMHD1 may help target L1 RNPs to SGs, its ability to degrade dNTPs, and so perhaps limit reverse transcription of non-LTR element RNAs, bears further investigation. The study of restriction factors that control retrotransposition has been dominated by cell culture retrotransposition assays: these may not always reflect in vivo reality. There are numerous mouse models with mutations in putative restriction factors that can now be screened by HT sequencing for altered endogenous retrotransposition. Many of these mice have mutations in RNAi pathway proteins; others are models for cancer and disease. For example, KO mice exist for most of the genes implicated in Aicardi Goutières Syndrome [513].

Some anti-viral defense strategies that are part of the intrinsic immune system of the cell have been coopted to inhibit non-LTR retrotransposition; the converse is likely also true. The coevolution of strategies by the cell to enforce and in turn by a retroelement to evade restriction perpetuates a molecular arms race. It has been proposed, for example, that the diversity of KRAB-ZFPs arose from their functions in silencing a diverse range of TEs [380]. The ability of retroelements to counter host restriction systems will determine which new subfamilies assume active dominance in a genome.

So far, the study of retrotransposon suppression has yielded surprising insights into gene regulation, epigenetics, DNA repair, and RNA interference. Intriguingly, some factors restrict both retroviruses and retrotransposons but by mechanisms that differ. It is logical, therefore, to study retrotransposon restriction factors as a means of gleaning new insights into viral control. For example, insights down the road may foster a better understanding of retroviral latency and perhaps contribute to its treatment. The continued presence of a reservoir of silenced HIV-1 proviral DNA integrated in the genomes of CD4+ T cells is a major obstacle to eradication of the virus in AIDs patients. Strategies under investigation attempt to overcome innate proviral repression, and then use standard antiretroviral therapy to kill cells with reactivated virus. Trials to date have had limited success and new insights are needed [514].

In cancers, widespread demethylation promotes retroelement transcription, and one would presume increased mobilization as well. Interestingly, to date bulk tissue sequencing efforts have detected new insertion events almost exclusively in tumors of epithelial cell types. To accurately assess the extent and tissue-specificity of retrotransposition, concerted efforts are needed to sequence many single cells from a large number of cell types (normal and cancerous) from many individuals. These efforts will help us to determine the extent to which ongoing insertions are drivers or passengers of cancer and disease. They will tell us if retrotransposition significantly influences brain diversity. To expedite these efforts, the field needs consensus concerning the best current protocols for capturing and identifying rare retrotransposon integrations in bulk tissues or single cells. We need effective algorithms that map retrotransposon transcript sequences in HT RNA-Seq data to their genomic source loci, a difficult task at present. With these algorithms, we will better determine how retrotransposon transcription changes under different cellular conditions and how it may modulate expression of cellular genes and impinge upon cell health. It is likely the physiological consequences of altered retrotransposon expression will be found to far exceed those of increased genome insertions.

While the great mass of the mobilome may be junk, the majority is benign, some is toxic waste, and occasionally bits of treasure may be found.

## Abbreviations

AGS. Aicardi–Goutières syndrome; ds. double-stranded; ESC. embryonic stem cell; HERV. human endogenous retrovirus; HNRNP. heterogeneous nuclear ribonucleoprotein; HT. high-throughput; IAP. intracisternal A-particle; IFN. interferon; iPSC. induced pluripotent stem cell; IRES. internal ribosome entry site; kD. kilodalton; KO. knock-out; KRAB-ZFP. Krüppel-associated box domain-containing zinc finger protein; LINE. long interspersed element; LTR. long-terminal repeat; MEF. mouse embryonic fibroblast; miRNA. microRNA; MTase. methyltransferase; ORF. open reading frame; PB. processing (P-) body; PGC. primordial germ cell; piRNA. Piwi-interacting RNA; qPCR. quantitative polymerase chain reaction; RISC. RNA-induced silencing complex; RNP. ribonucleoprotein; RT, reverse transcriptase; SG. stress granule; SINE. short interspersed element; siRNA. small interfering RNA; spcDNA. small polydispersed circular DNA; ss. single-stranded; SVA. SINE-R, VNTR, and Alu; TPRT. target-primed reverse transcription; TSD. target site duplication; UTR. untranslated region.
